# Interplay between interleukin-6 signaling and the vascular endothelium in cytokine storms

**DOI:** 10.1038/s12276-021-00649-0

**Published:** 2021-07-12

**Authors:** Sujin Kang, Tadamitsu Kishimoto

**Affiliations:** Laborabory of Immune Regulation, Immunology Frontier Research Center, Osaka University, 3-1 Yamadaoka, Suita, Osaka, Japan

**Keywords:** Sepsis, Interleukins

## Abstract

Interleukin-6 (IL-6) plays a crucial role in host defense against infection and tissue injuries and is a bioindicator of multiple distinct types of cytokine storms. In this review, we present the current understanding of the diverse roles of IL-6, its receptors, and its signaling during acute severe systemic inflammation. IL-6 directly affects vascular endothelial cells, which produce several types of cytokines and chemokines and activate the coagulation cascade. Endothelial cell dysregulation, characterized by abnormal coagulation and vascular leakage, is a common complication in cytokine storms. Emerging evidence indicates that a humanized anti-IL-6 receptor antibody, tocilizumab, can effectively block IL-6 signaling and has beneficial effects in rheumatoid arthritis, juvenile systemic idiopathic arthritis, and Castleman’s disease. Recent work has also demonstrated the beneficial effect of tocilizumab in chimeric antigen receptor T-cell therapy-induced cytokine storms as well as coronavirus disease 2019 (COVID-19). Here, we highlight the distinct contributions of IL-6 signaling to the pathogenesis of several types of cytokine storms and discuss potential therapeutic strategies for the management of cytokine storms, including those associated with sepsis and COVID-19.

## Inflammation: Stopping the storm

Blocking interleukin-6, a signaling molecule known as a cytokine, may help in treatment of both chronic and acute inflammation. IL-6 is part of a healthy immune response, but is also implicated in chronic inflammatory diseases such as rheumatoid arthritis and in ‘cytokine storms’, when the inflammatory response spirals out of control. Sujin Kang and Tadamitsu Kishimoto at Osaka University in Japan have reviewed the roles IL-6 plays in chronic and acute inflammation, and how an antibody to the IL-6 receptor, tocilizumab, shows therapeutic potential. They report that tocilizumab successfully blocks IL-6 signaling and has beneficial effects against rheumatoid and juvenile systemic idiopathic arthritis, and other chronic inflammatory conditions. Tocilizumab has also been shown to suppress cytokine storms brought on by CAR-T cell therapy and COVID-19. These results pave the way for better treatments for inflammatory conditions.

## Introduction

Interleukin-6 (IL-6) is a prominent proinflammatory cytokine released during infection or tissue injury that contributes to both innate and adaptive immune responses^1,2^. IL-6 is expressed promptly by innate immune cells, such as macrophages, upon their detection of damage-associated molecular patterns (DAMPs) or pathogen-associated molecular patterns (PAMPs) as part of the host defense strategy to remove infected cells or damaged tissue^2,3^. Excessive IL-6 production can lead to the development of chronic inflammatory diseases, such as rheumatoid arthritis (RA), and hyperinflammation, such as cytokine storms^4^, which are hyperreactive immune responses that can occur in patients infected with pathogenic bacteria or viruses, including severe acute respiratory syndrome coronavirus 2 (SARS-CoV-2), as well as in patients with leukemia treated by engineered T-cell therapy^5,6^. During a cytokine storm, large quantities of diverse active immune mediators, including cytokines, chemokines, and some growth factors, are produced quickly, which contributes to the progression and severity of the associated diseases. Hyperreactive immune responses can be suppressed by treatment with an antibody targeting the IL-6 receptor (IL-6R)^7,8^. To date, both the blockade of IL-6 itself and IL-6R signaling-based therapies have been successfully applied in the treatment of several chronic inflammatory disorders, such as RA, systemic and polyarticular juvenile idiopathic arthritis (JIA), Castleman’s disease, and large vessel vasculitis^9^. In addition, the humanized anti-IL-6R antibody tocilizumab has been approved for the treatment of cytokine storms induced by chimeric antigen receptor (CAR) T-cell therapy. Moreover, numerous clinical trials are ongoing to evaluate the efficacy of tocilizumab for SARS-CoV-2 infection-induced cytokine storms. The inhibition of IL-6 signaling may be beneficial for the treatment of cytokine storms. In this review, we highlight the pathological role of IL-6 and its signaling in systemic acute inflammatory diseases, and we discuss the targeting IL-6 and/or IL-6R as a clinical therapeutic strategy for cytokine storms.

## IL-6 signaling and associated intracellular pathways

**Fig. 1:**
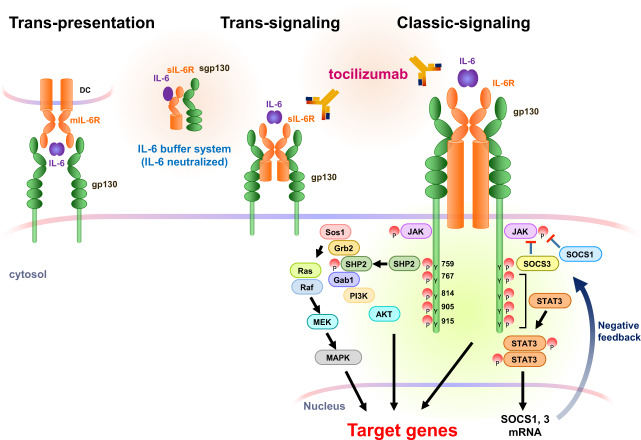
Three modes of IL-6R signaling and their associated intracellular pathways. To activate signaling, IL-6 requires two different receptors, IL-6R and gp130. IL-6 can bind to membrane-bound IL-6R (mIL-6R, classic signaling) or soluble IL-6R (sIL-6R, trans-signaling) or be presented by T cells through mIL-6R expressed on dendritic cells (trans-presentation). In all three modes of IL-6R signaling, a hexamer complex with gp130 is formed. IL-6 binding activates two main pathways: the JAK–MAPK pathway and JAK–STAT3 pathway. A tyrosine motif in the gp130 intracellular region exerts biological activities. To eliminate gp130 signaling activation, the expression of two cytokine receptor signaling inhibitors, SOCS1 and SOCS3, is induced by IL-6 signaling. Tocilizumab, an anti-human IL-6R monoclonal antibody, inhibits all three modes of IL-6R signaling, whereas soluble gp130 (sgp130) blocks only trans-signaling as a buffer system in the blood.

To initiate its pleiotropic effects, IL-6 activates signaling pathways by binding to both membrane-bound IL-6R (mIL-6R) or circulating soluble IL-6R (sIL-6R) and a second glycoprotein, gp130^10^. The pleiotropic activities of IL-6 are related to the ubiquitous expression of gp130, whereas IL-6R expression is restricted to a few cell types, including lymphocytes, monocytes/macrophages, and hepatocytes. IL-6 promotes its effects through three different signaling modes: classic signaling, trans-signaling, and trans-presentation (Fig. 1)^11^.

In classic signaling, IL-6 binds to a complex consisting of mIL-6R and gp130, subsequently transducing signals via activation of the JAK‒STAT3 pathway. In trans-signaling, IL-6 binds to sIL-6R present in serum and tissue fluid. sIL-6R is generated via the cleavage of mIL-6R by the proteases a disintegrin and metalloproteinase domain-containing protein 10 (ADAM10) and ADAM17^12^ or alternative IL-6R mRNA splicing^13^. In addition, the soluble form of gp130 (sgp130) present in serum can be incorporated into a complex consisting of IL-6/sIL-6R/sgp130^14^. sIL-6R has distinct functions depending on its concentration. At high concentrations of IL-6, sIL-6R binds to IL-6 to activate trans-signaling; this is inhibited by sgp130 as part of a buffering system by the formation of the IL-6/sIL-6R/sgp130 complex^15,16^, which regulates the IL-6 half-life in blood. Under high concentrations of IL-6 and sIL-6R, the IL-6 and sIL-6R complex binds to a homodimer of gp130 on cells that do not express IL-6R, such as endothelial cells. Recently, a third mode of IL-6 receptor signaling, observed only in dendritic cells, was identified^17^. In this so-called trans-presentation, the bound form of IL-6 and mIL-6R on dendritic cells is presented to T cells expressing gp130 on their surface. This alternative action of IL-6 signaling is an essential event for the priming of T helper 17 (Th17) cells.

The IL-6/sIL-6R‒gp130 complex can activate two main intracellular signaling pathways (Fig. 1). IL-6 promotes tyrosine phosphorylation in the gp130 cytoplasmic region by JAK kinases. JAK is constitutively bound to gp130. When JAK protein is phosphorylated by IL-6 stimulation, JAK activates STAT3 phosphorylation and induces homodimerization. The STAT3 homodimer functions as a transcription factor in the nucleus to induce the transcription of IL-6-responsive genes. The JAK kinase also activates the MAP kinase pathway through the binding of SHP2 to the gp130 phosphorylation site at tyrosine 759. This JAK‒SHP2‒MAPK pathway augments various transcriptional activities. The termination of gp130 activation is induced by STAT3-dependent IL-6R signaling via SOCS1 and SOCS3 expression^18^. SOCS1 forms a complex with activated JAK to repress its catalytic activity, whereas SOCS3 directly binds to phosphorylated gp130 at the tyrosine 759 site to attenuate JAK activation. Thus, SOCS family proteins partially control IL-6 receptor signaling via negative feedback (Fig. 1).

Several biological antibodies that inhibit IL-6, IL-6R, or related signaling factors have been developed and are therapeutically applicable for several chronic inflammatory diseases. The IL-6 antagonist siltuximab blocks only classic and trans-signaling of IL-6, whereas the anti-IL-6R antibodies tocilizumab and sarilumab inhibit all three modes of IL-6 signaling. Additionally, sgp130 can bind to complexes of IL-6–sIL-6R or IL-6–mIL-6R and exert inhibitory effects against both the trans-signaling and trans-presentation modes of IL-6 signaling^14,19^. Regarding intracellular signaling proteins, tofacitinib and baricitinib block the activities of JAK family proteins. Some inhibitory small molecules, but no antibodies, that block STAT3 activities have been developed. The clinical applications of these IL-6 signaling-targeted therapies in acute and systemic inflammatory diseases, such as cytokine storms, will be discussed below.

## Pathogenesis of IL-6 signaling in cytokine storms

Cytokine storm is a term encompassing immune disorders characterized by systemic inflammation, hyperinflammation and multiple organ failure (MOF)^20,21^. Mild symptoms generally include fever, fatigue, rash, and arthralgia. In severe cases, patients may have a high fever, increased levels of C-reactive protein (CRP), cytopenia, abnormal levels of coagulation parameters, disseminated intravascular coagulation (DIC), and damage to several organs, which can be fatal^6,22^. Cytokine storms can occur as a consequence of excessive cytokine production (e.g., with CAR T-cell therapy or hypersensitivity), ineffective host defense responses (e.g., with sepsis or Epstein–Barr virus (EBV)-associated hemophagocytic lymphohistiocytosis (HLH)), or a failed resolution of immune responses (e.g., with HLH). Cytokines play crucial roles in immune responses that help protect the host against pathogens. Thus, increased cytokine production controls some disseminated infections; however, sustained and excessive elevations in the levels of certain cytokines can cause negative systemic effects such as organ damage.

**Fig. 2:**
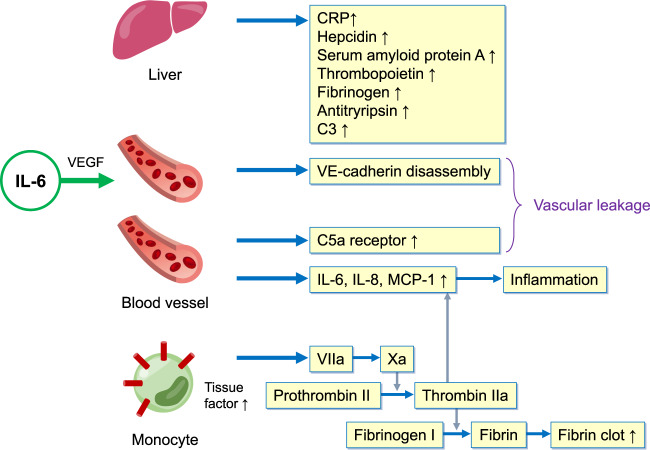
Modes of IL-6 activity in the acute phase of inflammation. In the liver, IL-6 acts on hepatocytes, where it promptly induces the expression of acute-phase proteins, such as CRP, serum amyloid A, antitrypsin, hepcidin, fibrinogen, thrombopoietin, and complement 3. In blood vessels, IL-6 can directly or indirectly act on vascular endothelial cells. IL-6 increases VE–cadherin disassembly through VEGF induction and C5a receptor expression, leading to vascular permeability. IL-6 directly stimulates vascular endothelial cells to produce proinflammatory cytokines via trans-signaling. In the context of coagulation cascade activation, IL-6 increases the expression of tissue factor on monocytes to increase fibrin clot formation. During this process, thrombin can also act on vascular endothelial cells to produce the proinflammatory cytokines IL-6, IL-8, and MCP-1.

A complex network of cell types, secreted cytokines, and signaling pathways is critically involved in the pathogenesis of cytokine storms. Elevated serum levels of IL-1, IL-6, IL-18, interferon (IFN)-γ, and tumor necrosis factor (TNF)-α are typical features of a cytokine storm. In particular, an elevated level of IL-6 is a hallmark of cytokine storms. This cytokine is an inflammatory mediator with roles in various acute inflammatory disorders^23–25^. In the setting of acute inflammation, IL-6 is released by several types of immune cells, such as macrophages and monocytes, and nonimmune cells, including vascular endothelial cells, mesenchymal cells, and fibroblasts, in response to stimulation by PAMPs/DAMPs or other cytokines, e.g., IL-1 or TNF-α^26^. Additionally, coagulation factors can trigger IL-6 production; for example, the administration of recombinant factor VIIa increased IL-6 levels in healthy subjects, and thrombin elicited the release of IL-6 from endothelial cells^27,28^. Rapidly produced IL-6 induces cells in several tissues, particularly the liver, to produce various acute-phase proteins, including CRP, hepcidin, serum amyloid protein A, thrombopoietin, fibrinogen, antitrypsin, and complement component C3^29,30^. IL-6 directly or indirectly elicits vascular endothelial injury via both VE–cadherin disassembly and increased C5a receptor expression on vascular endothelial cells, which lead to vascular leakage. Furthermore, IL-6 triggers activation of the coagulation cascade via the upregulation of tissue factors on monocytes^31^, subsequently promoting thrombin activation and accelerating fibrin clot formation (Fig. 2). The IL-6–sIL-6R complex also directly activates endothelial cells, which then release IL-6, IL-8, and MCP-1, recruiting immune cells and plasminogen activator inhibitor-1 (PAI-1) to promote coagulation cascade activation^32^.

Under healthy conditions, serum IL-6 is undetectable, whereas sIL-6R and sgp130 exist in molar excess. As described above, IL-6 binds to sIL-6R at nanomolar affinity, and the IL-6‒sIL-6R complex binds to sgp130 to exert its inhibitory activity at picomolar affinity, suggesting that the IL-6 concentration is regulated^33^. However, under inflammatory conditions, the serum sIL-6R concentration is increased, whereas sgp130 levels are comparable, leading to the activation of IL-6 trans-signaling by molar excess sIL-6R^33,34^. These scenarios lead to the systemic activity of IL-6 during inflammation. Of note, a single-nucleotide polymorphism (SNP) in the *IL6R* gene (rs7529229) has been found to be associated with a 2-fold increase in sIL-6R levels via the increased proteolytic cleavage of membrane-bound IL-6R, and this SNP was related to a low risk of coronary heart diseases^34,35^. Membrane-bound IL-6R mediates classical signaling in hepatocytes and some leukocytes, whereas sIL-6R induces trans-signaling in ubiquitous cell types expressing gp130, such as endothelial cells. This can be explained by the buffering of IL-6 activity via sIL-6R‒sgp130, which is more efficient for IL-6 trans-signaling mediated cell activation.

Notably, a monoclonal antibody that directly blocks IL-6R, tocilizumab, has dramatic effectiveness as a therapy for cytokine storms induced by various conditions, including Castleman’s disease, CAR T-cell-induced cytokine storm, and coronavirus disease 2019 (COVID-19)^9,20^.

## IL-6 signaling in Castleman’s disease

Castleman’s disease is a chronic lymphoproliferative disease that presents with multiple lymph node swelling, robust infiltration of mature plasma cells, sustained IL-6 production by germinal center B lymphocytes, and vascular hyperplasia^36^. Although the etiology of Castleman’s disease remains unclear, elevated levels of IL-6 are significantly correlated with symptom severity in this condition^37^. IL-6 has pleiotropic roles in the maturation of plasma cells and the production of acute inflammatory mediators and vascular endothelial growth factor (VEGF)^9,36^. In rodent experiments, IL-6 transgenic mice displayed features similar to those of patients with Castleman’s disease in that they have multiple lymph node swelling and follicular hyperplasia related to elevated IL-6 production^38^. Notably, human herpes virus-8 (HHV-8, also known as Kaposi’s sarcoma herpes virus) infection associated with multicentric Castleman’s diseases induces cytokine storms. HHV-8 encodes a homolog of human IL-6, referred to as viral IL-6 (vIL-6), which is produced by HHV-8-infected plasmablasts^39^. Transgenic mice expressing vIL-6 had serum levels of vIL-6 that were comparable with those of HHV-8-infected patients and developed splenomegaly, multifocal lymphadenopathy, hypergammaglobulinemia, and plasmacytosis^40^. Notably, transfer of the vIL-6 gene into IL-6-deficient mice abolished these symptoms, suggesting that endogenous IL-6 is a causative factor for the development of multicentric Castleman’s disease. In addition, patients with multicentric lymphadenopathy develop systemic inflammation in the context of cytopenia and potentially fatal MOF, which are driven by cytokine storms with high serum IL-6 levels^41^. Clinically, the surgical excision of a hyperplastic lymph node can improve the clinical symptoms and reduce the serum IL-6 level^42^. Furthermore, tocilizumab treatment in Castleman’s disease promptly attenuates all clinical symptoms and causes marked reductions in lymphadenopathy^43^. As such, tocilizumab was approved as an orphan drug for Castleman’s disease in Japan in 2005.

## IL-6 signaling in CAR T-cell therapy-induced cytokine storms

**Fig. 3:**
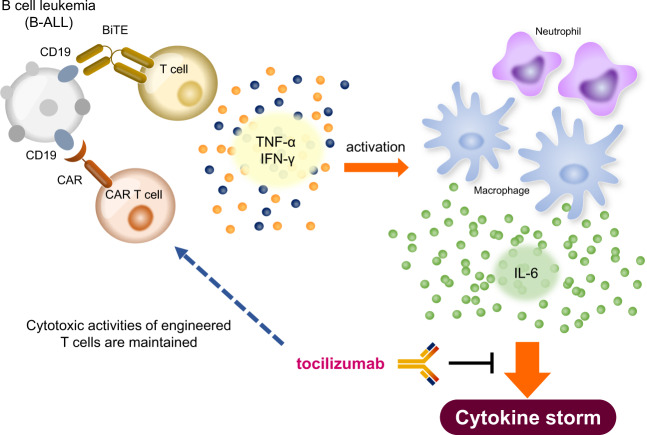
Mechanism of the inflammatory process in CAR T-cell therapy-induced cytokine storm. The activation of T cells or CAR T cells elicits the release of IFN-γ and TNF-α. These cytokines activate macrophages, subsequently inducing high levels of IL-6 production, which leads to a cytokine storm. Tocilizumab treatment can inhibit the development of a cytokine storm without blocking the cytotoxic activities of engineered T cells directed against B-cell leukemia (B-ALL).

In CAR T-cell therapy, T cells are engineered to recognize the CD19 antigen on B-cell lineage lymphoma cells, and the CAR is fused to the signaling region of the T-cell receptor^44^. Blinatumomab, a bispecific antibody that links CD3-positive T cells to a CD19 antigen, is also used as a therapy for lymphoma^45^. Both of these therapies cause the direct killing of lymphoma cells through cytotoxic T-cell activity and have shown high efficacy in relapsed and malignant B-cell lymphoma^46^. However, activated T cells release excessive levels of cytokines, such as IL-6, IL-10, and IFN-γ, which initiate a cytokine storm. Despite our limited understanding of the mechanism by which the immune response cascade is initiated and amplified to ultimate lead to a cytokine storm, identifying the major cytokines involved may help determine the pathogenesis of a cytokine storm. The complex composition of host immune cells, tumor cells, and administered CAR T cells forms an inflammatory circuit that is detrimental to patient health. As described above, the core cytokines elevated in patients with a cytokine storm are IL-6, IL-10, and IFN-γ. In the setting of CAR T-cell therapy, massive IFN-γ release by activated T cells or tumor cells triggers the development of a cytokine storm. IFN-γ can activate macrophages to produce excessive amounts of IL-6 and IL-10^47^. Subsequently, the high levels of IL-6 from macrophages activate IL-6R signaling in these same cells (referred to as classic signaling in Fig. 1) via a positive feedback loop (Fig. 3).

The first trial of tocilizumab resulted in successful outcomes for two patients with refractory acute lymphoblastic leukemia receiving CAR T-cell therapy^48^. Patients receiving CAR T-cell therapy can develop several serious symptoms, such as elevated cytokine levels, neutropenia, and B-cell aplasia. Treatment of these two patients with both the IL-6R inhibitor tocilizumab and the anti-TNFα antibody etanercept promptly attenuated the hyperinflammatory responses but did not interfere with the expansion of transplanted CAR T cells. Although some reports have suggested that CAR T cells themselves are a key factor for inflammatory cytokine release and T-cell activation as a positive feedback loop^49^, a number of studies in mice have indicated that the robust levels of proinflammatory cytokines involved in the development of a cytokine storm are mostly produced by macrophages. Indeed, blockade of IL-6 ameliorated hyperinflammatory responses in CAR T-cell-induced cytokine storms (Fig. 3)^50^.

The second trial was conducted in an acute lymphoblastic leukemia patient receiving blinatumomab who developed a cytokine storm; the patient’s serum levels of IL-6, IL-10, IL-8, MCP-1, IFN-γ, and soluble IL-2 receptor were strikingly elevated, while their serum levels of IL-1β, TNFα, and GM-CSF were normal. This serum cytokine pattern is similar to that found in cases of HLH^51^. After a single treatment with tocilizumab, the overreactive immune responses significantly decreased and nearly disappeared^23^. Large-scale clinical trials have since been performed in patients with serum IL-6 levels of over 1000 pg/ml, severe respiratory dysfunction, and activated coagulation factors such as D-dimer. Consistently, a single administration of tocilizumab markedly attenuated severe symptoms within 3 days of injection^52^. Therefore, IL-6 signaling inhibition is highly effective for improving severe clinical manifestations and organ dysfunction in most patients in whom the cytokine storm induced by CAR T-cell therapy is driven mainly by IL-6 signaling. Consequently, the FDA approved the usage of tocilizumab for treating CAR T-cell therapy-induced cytokine storms in 2017.

IL-6 appears to have a key role in mediating the critical symptoms of cytokine storms. Notably, severe cytokine storms that are characterized by coagulation cascade activation and vascular leakage are mediated by IL-6 trans-signaling^7,53^. Endothelial dysfunction contributes to the severity of cytokine storms by amplifying inflammatory reactions. Activated endothelial cells produce Ang-II and von Willebrand factor (vWF), and increased levels of these markers are present in the serum of patients with severe cytokine storms^54^. One patient with a severe cytokine storm who received CD19-targeted CAR T-cell therapy and subsequently died was found to have an extremely elevated IL-6 level caused by endothelial cell hyperactivation^55^. Notably, endothelial dysfunction may link cytokine storms and neurotoxicity. A recent clinical study reported that endothelial activation increases the risk of neurotoxicity after CAR T-cell therapy in patients^55^.

## IL-6 signaling in systemic inflammatory response syndrome (SIRS)

SIRS is recognized as a heterogeneous disease induced by microbial infections such as those in sepsis, tissue damage such as that seen in ARDS, or noninfectious causes, such as burns, ischemia, trauma, and graft-versus-host disease (GVHD)^5,56,57^. Despite extensive knowledge of the pathology of SIRS, it remains a problematic disease with high mortality and morbidity rates in patients who are very young, elderly, or critically ill^58^.

Sepsis is well defined as an acute and systemic inflammatory response to infection by bacteria, viruses (including influenza, dengue, and Ebola viruses), or parasites (such as Toxoplasma)^59^. The pathogenesis of sepsis-induced hyperinflammation is still unclear, and it is unknown which immune cells, cytokines, and chemokines are important for propagating the associated cytokine storm. In the early phase of sepsis, activated myeloid cells elicit striking elevations in various proinflammatory cytokines, such as IFN-γ, IL-1, IL-6, IL-8, IL-10, IL-18, MCP-1, TNF-α, and macrophage migration inhibitory factor (MIF)^60^. This hyperinflammation can lead to vascular endothelial injury and cardiac dysfunction, resulting in DIC and MOF, which together are fatal^61^. The severity of cytokine storms associated with severe sepsis, ARDS, and burns is likely caused by endothelial injuries; in patients with these conditions, elevated IL-6 levels are correlated with elevated levels of IL-8, MCP-1, and PAI-1^32,62,63^. Indeed, the in vitro activation of IL-6 trans-signaling in vascular endothelial cells, which express gp130 but not IL-6R, elicited marked increases in the levels of PAI-1, IL-6, IL-8, and MCP-1. This suggests that IL-6 trans-signaling in the endothelium shapes the proinflammatory cytokine network during a cytokine storm. Unfortunately, despite observations of improved mortality in murine sepsis models following blockade of TNF-α or IL-1 signaling, several clinical trials of TNF or IL-1 inhibitors failed to achieve the endpoint for patients with sepsis^64,65^.

Although IL-6 is a useful biomarker and diagnostic indicator of sepsis progression^66,67^, the administration of anti-IL-6 or anti-IL-6R therapy for the treatment of sepsis has not been studied because IL-6 is an important factor driving immune activation as a host defense against infection. However, accumulating results from animal experiments suggest that the inhibition of IL-6 or IL-6R is promising for treating infectious and noninfectious SIRS. In a murine cecal ligation puncture (CLP) model, the administration of anti-IL-6 antibody improved survival through the reduction of C5a expression^68^. In line with this, anti-IL-6 antibody injection reduced coagulation cascade activation in a chimpanzee endotoxin model^69^. However, the sustained inhibition of IL-6 signaling might result in worse outcomes because of an inability to eliminate invading microbes. Notably, the administration of soluble gp130-Fc (sgp130-Fc), which selectively inhibits IL-6 trans-signaling, improved mortality in a CLP model through the abrogation of epithelial cell apoptosis^70^, whereas IL-6 neutralization did not improve mortality in a sepsis model^71,72^. Collectively, accumulating evidence indicates that the inhibition of IL-6 trans-signaling is a promising therapy for sepsis. Because IL-6 influences host defense, blockade of IL-6 signaling silences inflammatory responses, which makes it difficult to assess the severity of infection. Therefore, the use of IL-6 signaling inhibition to treat sepsis should be very carefully considered.

## IL-6 signaling in COVID-19-induced cytokine storm

The ongoing outbreaks of infection by SARS-CoV-2, the causative pathogen of COVID-19, led to the declaration of a pandemic by the World Health Organization (WHO). COVID-19 is a life-threatening disease with a high mortality rate worldwide. The symptoms of COVID-19 are broad, ranging from mild symptoms and pneumonia to ARDS and MOF. Severe cases of COVID-19, associated with high mortality, often occur in persons who are elderly or have underlying diseases and are consequently unable to control virus expansion. Although the mechanisms of COVID-19-induced cytokine storm and lung injury are still poorly understood, some reports have indicated that the overproduction of proinflammatory cytokines, such as IL-6, IL-1β, and TNFα, in COVID-19 patients has a pattern that is similar to that seen in a cytokine storm, which is known to lead to an increased risk of vascular permeability, DIC, and respiratory failure^73,74^. Therefore, anti-cytokine reagents have potential as therapeutic agents for COVID-19, but consideration of their use must be balanced with the need to maintain a proper inflammatory response for pathogen elimination.

**Fig. 4:**
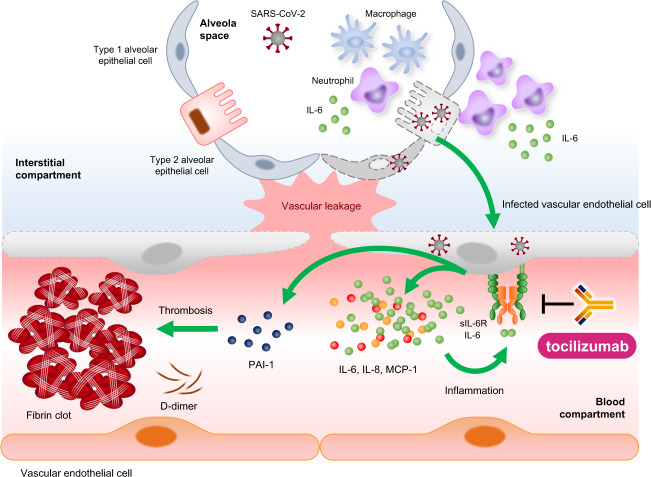
Mechanism of the inflammatory process in COVID-19-induced cytokine storm. The pathophysiological features of COVID-19 in the lungs and vasculature are illustrated. SARS-CoV-2 infection induces a loss of vascular integrity, activation of coagulation, and amplification of inflammation through IL-6 trans-signaling. Tocilizumab may act on endothelial IL-6 trans-signaling and attenuate the symptoms of cytokine storms due to COVID-19.

Recently, endothelial cells were suggested as a therapeutic target in COVID-19 because emerging data suggest that endothelial cell dysfunction contributes to the severity of COVID-19 by causing vascular injuries, promoting the coagulation cascade, inducing endotheliitis, and recruiting activated immune cells^75^. Endothelial cells maintain vascular integrity and barrier function under normal conditions, and they also prevent inflammation via the production of anticoagulation factors and blood clot-lysing enzymes and the expression of glycocalyx, which has anticoagulant properties^75^. In regard to viral infections such as SARS-CoV-2, severe lung damage caused by endotheliitis and coagulation pathway activation was found in patients with severe COVID-19^76^. Mechanistically, pulmonary complications in patients with COVID-19 have several causes. First, SARS-CoV-2 directly infects endothelial cells in several organs, as observed in deceased patients. Viral infection induces endothelial cell dysfunction and lysis, resulting in endotheliitis, and subsequently mediates the potential development of DIC and eventual death^75^. Second, COVID-19-associated thromboinflammation is frequently found and is a major cause of mortality in critically ill patients. Laboratory analyses have found evidence of coagulopathy in the form of high serum levels of D-dimer and thrombocytopenia in hospitalized patients with COVID-19^74,76,77^. In line with this, numerous markers of endothelial injury, such as vWF and PAI-1, are particularly high in patients with COVID-19 who are admitted to the intensive care unit, which indicates that the levels of vWF and PAI-1 are highly correlated with COVID-19 severity^78,79^. Finally, elevated serum levels of proinflammatory cytokines, such as IL-6 and IL-1β, in critically ill patients with COVID-19 were found to be associated with abnormal coagulation parameters, which are caused by endothelial dysfunction. Notably, serum IL-6 levels were highly correlated with fibrinogen levels in patients with COVID-19^80^. Consistently, our previous study highlighted many features of endotheliopathy, such as elevated levels of PAI-1, which were correlated with serum IL-6 levels in not only patients with severe COVID-19 but also those with other types of cytokine storms^32^. Although proinflammatory cytokines, including IL-6, were elevated to moderate levels in critically ill patients, PAI-1 levels were extremely high, reaching levels similar to those seen in patients with bacterial sepsis or ARDS. Thus, these mechanisms can lead to endothelial injury and vascular leakage (Fig. 4).

The first reports demonstrating an association between serum IL-6 levels and COVID-19 severity came from Wuhan at the beginning of the COVID-19 pandemic^81^. Critically ill patients with COVID-19 exhibited pneumonia-like symptoms and extremely elevated serum IL-6 levels, similar to those seen in cases of HLH. At the beginning of the COVID-19 pandemic, clinicians in Wuhan used off-label tocilizumab to treat hospitalized patients with severe COVID-19. This first trial of tocilizumab in critically ill patients with COVID-19 resulted in successful outcomes; it improved the clinical symptoms and CRP concentrations of the treated patients^81^. Consistently, our off-label trial of tocilizumab in critically ill patients with COVID-19 indicated that tocilizumab treatment significantly decreased serum PAI-1 and CRP levels and improved several clinical parameters^32^. These data suggest that the prevention of endothelial injury via IL-6 signaling inhibition is crucial for preventing increased COVID-19 severity characterized by cascade coagulation activation and microvascular thrombosis.

Recently, it was reported that dexamethasone reduced mortality in severe COVID-19 patients who required supplemental oxygen^82^. This result suggests that achieving additional improvements in clinical outcomes may require more specific immunomodulatory agents^82,83^. Although several clinical trials on the use of tocilizumab to treat COVID-19 have been conducted, the completed randomized controlled trials have been inconclusive^84–87^. The first randomized trial of tocilizumab, the COVACTA trial, failed to meet its primary endpoint of improvement in clinical parameters. Despite tocilizumab treatment failing to improve mortality, it was found to shorten the length of ICU stay compared with placebo treatment, which is a clinically meaningful result^88^. However, tocilizumab treatment was not associated with COVID-19 improvement in moderately ill patients^87^. Another randomized trial, the EMPACTA trial, which included only severe cases of COVID-19, found that tocilizumab treatment had the beneficial effect of reducing the need for mechanical ventilation but did not improve the overall mortality^86^. Notably, the REMAP-CAP trials conducted in the United Kingdom, which assessed the effectiveness of blockade of IL-6R signaling using tocilizumab or another anti-IL-6R antibody (sarilumab), recently reported the beneficial effect of a combination of either tocilizumab or sarilumab with standard of care^89^. More recently, the RECOVERY trial, a large randomized controlled trial of tocilizumab conducted in critically ill adult patients who exhibited systemic inflammation and high CRP levels and needed oxygen support, reported that tocilizumab treatment improved survival and reduced the chance of disease progression to a state requiring invasive mechanical ventilation^90^. Although the RECOVERY trial results are preliminary, they support the use of tocilizumab to treat COVID-19. Overall, the available clinical evidence suggests a benefit of IL-6R antagonists as therapeutics in patients with COVID-19.

## Conclusion

IL-6, an important indicator of cytokine storms, has been found to regulate several aspects of vascular homeostasis and cellular inflammation. Emerging evidence indicates that IL-6 signaling inhibition is a beneficial clinical strategy for the treatment of various inflammatory disorders, including RA, Castleman’s disease, and CAR T-cell therapy-induced cytokine storms. In analyses of the pathological mechanisms of COVID-19, critically ill patients with COVID-19 have shown significantly elevated levels of IL-6, and tocilizumab treatment was found to be beneficial in these patients.

Because the infection is a major side effect associated with tocilizumab treatment, the development of an IL-6 inhibitor with a short half-life for use in the treatment of pathogen-induced cytokine storms, such as those cause by sepsis, is desirable. Moreover, endothelial homeostasis acts as a gatekeeper of immune responses, as the inhibition of IL-6 trans-signaling in endothelial cells dampens the propagation of cytokine storms associated with sepsis, ARDS, burns, and severe cases of COVID-19. In the setting of hyperinflammation, it remains unclear why IL-6 expression is sustained and how the inhibition of IL-6 signaling has positive efficacy in patients with a cytokine storm. Although the therapeutic potential of mitigating IL-6 signaling-induced vasculature injury during cytokine storms requires further validation, the detailed mechanisms linking IL-6 receptor signaling to vascular leakage and coagulation cascade activation should be identified to uncover the pathogenesis of cytokine storms.
